# Prevalence and time trends of refractive error in Chinese children: A systematic review and meta-analysis

**DOI:** 10.7189/jogh.11.08006

**Published:** 2021-07-17

**Authors:** Yi Tang, Aiming Chen, Minjie Zou, Zhenzhen Liu, Charlotte Aimee Young, Danying Zheng, Guangming Jin

**Affiliations:** 1 State Key Laboratory of Ophthalmology, Zhongshan Ophthalmic Center, Sun Yat-Sen University, Guangzhou, China; 2 Zhongshan School of Medicine, Sun Yat-sen University, Guangzhou, China; 3 Department of Pharmacy, The Fifth Affiliated Hospital of Sun Yat-Sen University, Zhuhai, China; 4 Department of Ophthalmology, Third Affiliated Hospital, Nanchang University, Nanchang, China

## Abstract

**Background:**

To investigate the prevalence and time trends of refractive error (RE) among Chinese children under 18 years old.

**Methods:**

PubMed, Embase, Web of Science were searched for articles that estimated prevalence of RE in Chinese children. Data of identified eligible studies was extracted by two investigators independently. Pooled prevalence of RE and its 95% confidence interval (95% CI) and the time trends of RE were investigated using Meta-analysis methods.

**Results:**

Of the 41 studies covering 1 051 784 subjects, the pooled prevalence of myopia, high myopia, hyperopia and astigmatism in Chinese children was 38.0% (95% confidence interval (CI) = 35.1%-41.1%), 2.8% (95% CI = 2.3%-3.4%), 5.2% (95% CI = 3.1%-8.6%) and 16.5% (95% CI = 12.3%-21.8%), respectively. Subgroup analysis show that children living in urban were at higher risk of RE. Prevalence of myopia and hyperopia were higher in Northern China compared with Southern China and high myopia and astigmatism were higher in Hong Kong, Macau and Taiwan than in mainland China. Regression analysis showed an upward trend in myopia and hyperopia and a downward trend in high myopia and astigmatism with years.

**Conclusions:**

The prevalence of RE is higher in urban areas than in rural for Chinese children. The much higher prevalence of myopia and astigmatism in China compared with foreign countries indicates the important role played by environment and genetic factors. Considering the large magnitude of refractive errors, much more attention should still be paid to RE prevention and treatment strategy development in China.

Refractive error (RE) has been one of the most common eye disorders among children and adolescents and one of the major public health concerns in the world. It has been reported that 42% of visual impairments are caused by RE globally [1]. RE have profound effects on children, for not only will it increase the possibility of pathologic ocular changes such as myopic macular degeneration and retinal detachment, which could lead to irreversible blindness, but it also has a great impact on psychosocial well-being for children, which can limit their educational outcomes and educational opportunities [2-4].

In East Asia, the high prevalence of RE has been a major public health concern. For urban areas of these countries, about 80% of the adolescents in high school are myopic, while 10%-20% of them suffer from high myopia [5]. Also, it is reported that the prevalence of hyperopia and astigmatism in Asian children was 4.6% and 14.9% respectively [6]. Moreover, striking evidence suggests a growing trend of RE prevalence especially among young East Asians [7,8]. It is expected that by 2050, 4758 million people will be myopic and 938 million people will suffer from high myopia globally [9]. China, the most populous country in the world that accounting for one fifth of world population, has had a high prevalence of RE during the past decades and possessing a large number of RE patients [6-8,10].

Considering the impact of RE and its high prevalence, it is undeniable that there is great value in further understanding the epidemiology of RE for the purposes of policy making. Particularly, policy of myopia prevention and control has been a hotspot in the field of public health since the rapid rise of prevalence of myopia in China. Although numerous population-based or school-based studies and meta-analysis have been performed to evaluate the prevalence of RE in China, most of them focus on myopia and high myopia [10-13], which does not shed light on the magnitude and time trend of total RE, especially hyperopia and astigmatism, among the young Chinese population and there is a lack of study reporting the epidemiologic characteristics of RE as a whole.

Considering an overall estimate of the magnitude and its time trends of RE is important for RE prevent and control, we performed this meta-analysis and comprehensive systematic review to evaluate the prevalence of refractive errors, time trends, and its sub-classifications among children in China, which might provide useful information for appropriate preventive strategies to reduce the disease burden caused by SE in China and beyond.

## METHODS

### Search strategy

The protocol of the meta-analysis was registered in PROSPERO website (University of York, United Kingdom) with a registration number of CRD42020197708. In order to extract articles providing prevalence data of refractive errors in Chinese children, bibliographic databases including PubMed, EMBASE, and Web of Science were searched with different combinations of words including

1. Population: “China”, “Chinese”, “Taiwan”, “Macau”, “Macao”, “Hong Kong”
2. Outcome: “refractive errors”, “myopia”, “astigmatism”, “hyperopia”
3. Study design: “Prevalence”, “Epidemiology”, “epidemiology”, “prevalence”, “incidence”

The search was conducted by two investigators (TY, ZMJ) independently with the final search date of July 28, 2019.

### Study selection

After the search, 4240 articles were identified. 1641 duplicate articles were removed. The selection was conducted by two investigators independently with the following criteria:

The inclusion criteria were as follows: 1) school-based studies or population-based studies with clearly defined sampling strategies; 2) studies reporting the prevalence of refractive errors in Chinese children younger than 18 years old; 3) studies with a clear definition of refractive errors; 4) sample size of at least 1000 subjects. Studies with sample size less than 1000 were excluded because it’s age-defined subgroups would be too small for a reliable assessment of the prevalence of refractive errors.

The exclusion criteria were as follows: 1) hospital-based or clinical-based surveys; 2) conducted only in a single school; 3) using visual acuity as the measurement for refractive errors; 4) missing or incomplete data; 5) obvious limitations in their statistical analysis or design; 6) different studies based on the same population without providing additional information.

### Data extraction

The searches were limited to English language literature only. After the selection procedure, 41 articles that met the inclusion criteria were carefully reviewed by two investigators (TY, ZMJ). The extracted data of these articles are listed as follows:

1. Characteristics of the study: author, study year, design of study, refraction with or without mydriatics.
2. Characteristics of the studied population: sample size, age range, district and region (urban or rural) of the sample, percentage of female subjects.
3. Prevalence data: definition and prevalence of refractive errors.

### Statistical analysis

Pooled prevalence of refractive error and its 95% confidence interval (95% CI) was estimated. Subgroup analyses were conducted for potential difference in region and study year. The I-square test was performed to estimate the heterogeneity of the included studies (<50% indicates low heterogeneity, and >50% presents high degree of heterogeneity). When the I-square test suggested a high degree of heterogeneity, a random effect model was used, otherwise a fixed effect model was used.[10] To access the publication bias of these studies, Egger’s tests and Begg’s tests were performed and the significance level was set at *P* < 0.05 (2-tailed). Funnel plots were also performed for publication bias when more than 10 studies were involved in the meta-analysis. Sensitivity analysis of studies included in myopia, high myopia, hyperopia, astigmatism were conducted. Time trends of refractive errors were also investigated by meta regression. This meta-analysis was performed with the Comprehensive Meta-Analysis Software V.2 (Biostat, Englewood, New Jersey, USA).

## RESULTS

**Figure 1:**
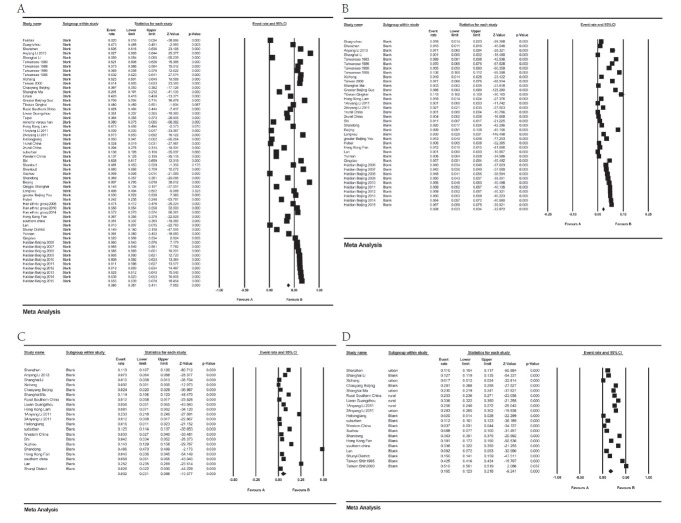
Flowchart of the study selection process.

**Figure 1** shows the process of literature selection. 4240 records were identified by literature research. After the selection, 41 studies including 1 051 784 subjects were included for qualitative synthesis [11-51]. Among these studies, 15 studies were conducted in Northern China including 196 547 subjects (18.7%) [11,13,17,22,23,25,28,33,34,37,38,40,41,48,49]; 19 studies with 98885 participants (9.4%) were conducted in Southern China [14-16,18,19,21,24,29-32,35,36,39,42,43,45-47]; 6 studies including 89 213 subjects (8.5%) were launched in Hong Kong, Macao and Taiwan (HMT), [12,20,26,27,44,50], and 1 multicenter study included 667139 individuals (63.4%) [51]. The basic characteristics of these studies are shown in **Table 1** and the prevalence and definitions of RE are given in **Table 2**.

**Table 1:** The basic characteristics of included studies

Author	Study year	District	Region	Design of study	Sample size	Age(mean or range)	Girls	Refraction	Cyclopiegia
Guo et al. [14]	NA	SC	U and R	SB	5182	3-6	NA	AU	yes
Guo et al. [15]	2014	SC	U	SB	3055	7-15	48.3	AU	yes
Han et al. [16]	2015	SC	U	SB	8662	5-16	45.17	AU	yes
Li et al. [22]	2013	NC	U	SB	1839	12.9-17.6	51.6	AU	yes
Li et al. [24]	2013	SC	U	SB	7166	4-6	46.8	AU	no
Lin et al. [26]	1983	HMT	U and R	SB	4125	7-18	NA	AU	yes
	1986	HMT	U and R	SB	10 500	7-18	NA	AU	yes
	1990	HMT	U and R	SB	8667	7-18	NA	AU	yes
	1995	HMT	U and R	SB	11 178	7-18	49.2	AU	NA
Congdon et al. [43]	2007	SC	R	SB	1892	11.4-17.1	51.2	AU	yes
Lin et al. [27]	2000	HMT	U and R	SB	10 889	7-18	48	AU	yes
Lyu et al. [28]	2011	NC	U	SB	4249	5-14	48.2	AU	yes
Ma et al. [29]	2013	SC	R	SB	5532	3-10	45.3	AU	yes
Qian et al. [33]	2017	NC	U	SB	8683	6-18	45.7	AU	no
Guo et al. [11]	2016	NC	U and R	SB	35 745	6-18	48.9	AU	no
Han et al. [17]	2013	NC	R	SB	2147	11-15	48.1	AU	NA
Li et al. [25]	2008-2009	NC	R	PB	1675	5-18	46.1	AU	yes
Pan et al. [30]	2016	SC	R	SB	2432	mean7.7	44.8	AU	yes
	2016	SC	R	SB	2346	mean13.8	48.3	AU	yes
Pi et al. [31]	2006-2007	SC	R	PB	3070	6-15	47.5	RE	yes
Pi et al. [32]	2006	SC	R	PB	3079	6-15	47.5	RE	NA
Shi et al. [34]	NA	NC	U	SB	2046	7 to 12	46.1	AU	no
Wang et al. [35]	2009	SC	U and R	SB	1235	13-15	51.2	AU	NA
	2009	SC	U and R	SB	1183	16-18	42.8	AU	NA
Wang et al. [36]	2011	SC	U	SB	2255	2-6.7 (24-80 months)	44.7	RE	yes
Wu et al. [37]	NA	NC	R	SB	6026	4-18	47.1	AU	yes
Wu et al. [38]	NA	NC	U and R	SB	4677	15-18	53.7	AU	no
Hsu et al. [20]	2013	HMT	U and R	PB	11 590	8 (grade 2)	47.1	AU	yes
Hu et al. [21]	2014	SC	R	SB	10 037	9-12	47.7	AU	no
Lam et al. [12]	2005-2010	HMT	U	SB	2651	5-15	46.8	AU	no
Li et al. [23]	2011-2012	NC	U	SB	2893	5.7-9.3	42.2	AU	yes
	2011-2012	NC	U	SB	2267	10.0-15.9	50	AU	yes
Xia et al. [39]	2009	SC	R	SB	3517	7-11	44.2	AU	yes
Yang et al. [40]	2015	NC	U and R	SB	61 036	7-18	48.7	AU	no
Zeng et al. [42]	2017	SC	U and R	SB	16 955	6-10	44.2	RE	yes
Zhao et al. [49]	1998	NC	R	PB	5884	5-15	48.9	AU	yes
Qian et al. [47]	2014	SC	R	SB	7681	5-16	49.3	AU	yes
Dong et al. [51]	2005	M	U and R	PB	235 505	7-18	49.8	NA	NA
	2010	M	U and R	PB	216 474	7-18	50	NA	NA
	2014	M	U and R	PB	215 160	7-18	50	NA	NA
Fan et al. [44]	1998-2000	HMT	U	SB	7560	6-15	49.5	AU	yes
He et al. [19]	2002-2003	SC	U	PB	4364	5-15	48.4	AU and RE	yes
Lan et al. [46]	2009	SC	U and R	SB	2478	3-6	47.2	AU and RE	yes
Sun et al. [48]	2015-2016	NC	U	SB	4890	10-15	48.3	AU	yes
Shih et al [50]	1995	HMT	U and R	SB	11 175	7-18	NA	AU	yes
	2000	HMT	U and R	SB	10 878	7-18	NA	AU	yes
Li et al. [13]	2006	NC	U	SB	3657	14-16	52.37	AU	yes
	2007	NC	U	SB	3615	14-16	52.42	AU	yes
	2008	NC	U	SB	3662	14-16	52.81	AU	yes
	2009	NC	U	SB	3697	14-16	50.42	AU	yes
	2010	NC	U	SB	3897	14-16	52.45	AU	yes
	2011	NC	U	SB	3784	14-16	49.1	AU	yes
	2012	NC	U	SB	3816	14-16	54.09	AU	yes
	2013	NC	U	SB	3787	14-16	52.05	AU	yes
	2014	NC	U	SB	3833	14-16	53.48	AU	yes
	2015	NC	U	SB	3676	14-16	51.8	AU	yes
He et al. [45]	2002-2003	SC	U	PB	4364	5-15	48.5	RE	yes
You et al. [41]	2012	NC	U and R	PB	15 066	7-18	51.6	AU	no
He et al. [18]	2005	SC	R	SB	2400	13-17	49.1	AU	yes

SC – Southern China, NC – Northern China, HMT – Hong Kong, Macao and Taiwan, M – multiple center, U – urban, R – rural, SB – school-based, PB – population-based, AU – auto refraction, RE – retinoscopy

**Table 2:** Prevalence of refractive errors in the included studies

Author	Myopia		High myopia		Hyperopia		Astigmatism	
**Definition**	**Prevalence**	**Definition**	**Prevalence**	**Definition**	**Prevalence**	**Definition**	**Prevalence**
Guo et al. (NA)* [14]	SE≤-0.5D	1.95	NA	NA	NA	NA	NA	NA
Guo et al. (2014) [15]	SE≤-0.5D	47.3	SE≤-6.0D	1.8	NA	NA	NA	NA
Guo et al. (2016) [11]	SE≤-0.5D	70.9	SE≤-6.0D	8.6	NA	NA	NA	NA
Han et al. (2015) [16]	SE≤-0.25D	62.6	SE≤-6.0D	1.3	SE≥0.5D	11.3	SE≥0.5D	11
Han et al. (2013) [17]	SE≤-0.75D	48.02	SE<-6.0D	11.5	NA	NA	NA	NA
Li et al. (2013) [22]	SE≤-0.5D	82.7	NA	7.1	SE≥0.5D	7.5	NA	NA
Li et al. (2013) [24]	SE≤-1.0D	5.9	SE≤-6.0D	0.1	SE≥2.0D	1	SE≥1.0D	12.7
Li et al. (2011, 2012) [23]	SE≤-0.5D	3.9	SE≤-6.0D	0.1	SE≥2.0D	23.3	SE≥0.75D	25.6
	SE≤-0.5D	67.3	SE≤-6.0D	2.7	SE≥2.0D	1.2	SE≥0.75D	28.3
Li et al. (2008) [25]	SE≤-0.5D	5	NA	NA	SE≥0.5D	1.6	SE≥0.75D	2
Lin et al. (1983, 1986, 1990, 1995) [26]	SE<-0.25D	62.1	SE<-6.0D	8.9	NA	NA	NA	NA
	SE<-0.25D	57.5	SE<-6.0D	7	NA	NA	NA	NA
	SE<-0.25D	56.8	SE<-6.0D	5.5	NA	NA	NA	NA
	SE<-0.25D	63.2	SE<-6.0D	10.6	NA	NA	NA	NA
Lin et al. (2000) [27]	SE<-0.25D	61.4	SE<-6.0D	7.1	NA	NA	NA	NA
Lyu et al. [28]	SE≤-0.5D	36.7	NA	NA	SE≥2.0D	2.4	SE≥1.0D	28.1
Qian et al. (2017) [33]	SE≤-0.75D	42.8	NA	NA	NA	NA	NA	NA
Qian et al. (2014) [47]	SE<-0.5D	39.1	SE<-6.0D	0.6	NA	NA	NA	NA
Hsu et al. [20]	SE≤-0.5D	36.4	NA	NA	NA	NA	NA	NA
Hu et al.[21]	SE≤-0.5D	8	NA	NA	SE≥2.0D	NA	SE≥0.75D	NA
Lam et al.[12]	SE<-0.5D	47.5	SE<-6.0D	1.8	SE>0.5D	8.1	NA	NA
Pan et al.[30]	SE<-0.5D	2.4	SE<-6.0D	0.1	NA	NA	NA	NA
	SE<-0.5D	29.4	SE<-6.0D	0.4	NA	NA	NA	NA
Pi et al.(2006-2007)[31]	SE≤-0.5D	13.75	NA	NA	SE≥1.5D	12.51	SE≥0.5D	11.17
Pi et al.(2006)[32]	SE≤-0.5D	13.7	NA	NA	SE≥2.0D	3.3	SE≥1.0D	3.7
Shi et al.[34]	SE≤-0.5D	63.8	SE<-6.0D	1.1	SE>0.5D	4.2	NA	NA
Ma et al.[29]	SE≤-0.5D	20.1	SE≤-6.0D	0.3	SE≥2.0D	11.4	SE≤-1.0D	23
Congdon et al.[43]	SE<-0.5D	62.3	SE<-6.0D	1.9	SE≥2.0D	0.2	SE>0.75D	1.7
Wu et al.(NA) [37]	SE≤-0.5D	36.9	SE≤-6.0D	2	SE>0.5D	48.6	SE≥0.75D	36.3
Wu et al.(NA) [38]	SE≤-1.0D	80.7	SE≤-6.0D	9.9	NA	NA	NA	NA
Wang et al.(2009)[35]	SE≤-0.75D	48.07	NA	NA	NA	NA	NA	NA
	SE≤-0.75D	68.28	NA	NA	NA	NA	NA	NA
Wang et al.(2011)[36]	SE≤-1.0D	0.9	NA	NA	SE≥2.0D	14.3	SE≥1.0D	8.8
Xia et al.[39]	SE<-0.5D	14.5	NA	NA	NA	NA	NA	NA
Yang et al.[40]	SE≤-0.5D	49.8	SE≤-6.0D	3	NA	NA	NA	NA
You et al.[41]	SE≤-1.0D	53	SE≤-6.0D	4.3	NA	NA	NA	NA
Zeng et al.[42]	SE≤-0.5D	24.15	SE<-6.0D	0.64	NA	NA	NA	NA
Dong et al.[51]	SE≤-0.5D	47.4	NA	NA	NA	NA	NA	NA
	SE≤-0.5D	55.6	NA	NA	NA	NA	NA	NA
	SE≤-0.5D	57.2	NA	NA	NA	NA	NA	NA
Fan et al.[44]	SE≤-0.5D	36.71	SE≤-6.0D	1.19	SE≥2.0D	4	SE≥1.0D	18.1
He et al.(2002) [45]	SE≤-0.5D	35.1	NA	NA	SE≥2.0D	5.8	SE≥0.75D	33.6
He et al.(2005) [18]	SE≤-0.5D	42.4	NA	NA	SE≥2.0D	1.2	SE≥0.75D	25.3
He et al.(2002) [19]	SE≤-0.5D	35.1	NA	NA	SE>2.0D	5.8	SE≥0.75D	33.6
Lan et al. [46]	SE≤-0.5D	1	SE≤-6.0D	0.1	SE≥2.0D	25.2	SE≥1.5D	8.2
Zhao et al. [49]	SE≤-0.5D	14.9	NA	NA	SE≥2.0D	2.6	SE≥0.75D	15
Shih et al. [50]	NA	NA	NA	NA	NA	NA	SE≥0.5D	42.5
	NA	NA	NA	NA	NA	NA	SE≥0.5D	51
Sun et al. [48]	SE<-0.5D	52.02	SE≤-6.0D	5.7	NA	NA	NA	NA
Li et al. (2006, 2007, 2008, 2009, 2010, 2011, 2012, 2013, 2014, 2015) [13]	SE<-0.5D	55.95	SE<-6.0D	3.96	NA	NA	NA	NA
	SE<-0.5D	56.49	SE<-6.0D	4.18	NA	NA	NA	NA
	SE<-0.5D	58.47	SE<-6.0D	4.75	NA	NA	NA	NA
	SE<-0.5D	60.54	SE<-6.0D	4.98	NA	NA	NA	NA
	SE<-0.5D	60.79	SE<-6.0D	5.52	NA	NA	NA	NA
	SE<-0.5D	61.13	SE<-6.0D	5.89	NA	NA	NA	NA
	SE<-0.5D	61.84	SE<-6.0D	5.92	NA	NA	NA	NA
	SE<-0.5D	62.77	SE<-6.0D	6.02	NA	NA	NA	NA
	SE<-0.5D	63.84	SE<-6.0D	6.42	NA	NA	NA	NA
	SE<-0.5D	65.48	SE<-6.0D	6.69	NA	NA	NA	NA

SE – spherical equivalent, D – diopters, NA – not available

*Study year are shown in the brackets for authors with the same name.

### Prevalence of myopia and high myopia

**Figure 2:**
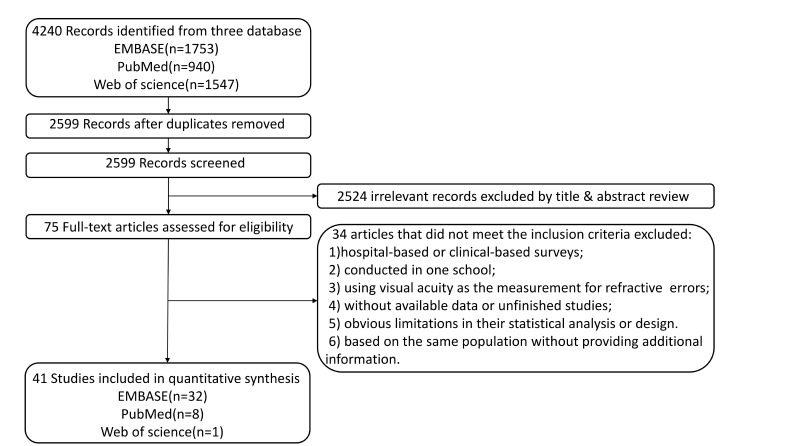
Meta-analysis showing the pooled prevalence of myopia (Panel A), high myopia (Panel B), hyperopia (Panel C) and astigmatism (Panel D) in Chinese children.

As is shown in **Table 3** and **Figure 2****,** Panel A, the pooled prevalence of myopia was 38.0% (95% CI = 35.1%-41.1%). Urban children had a significantly higher prevalence of myopia than rural children: 51.2% (95% CI = 50.8%-51.5%) vs 27.1% (95% CI = 26.7%-27.5%); *P* < 0.001. Additionally, pooled prevalence in HMT and Northern China were similar: 53.0% (95% CI = 52.6-53.4%) vs 55.1% (95% CI = 54.9%-55.3%), while Southern China hadhas the lowest prevalence: 31.4% (95% CI = 31.0%-31.7%), *P* < 0.001. The subgroup analysis of study year shows that there was a higher prevalence before the year 2000: 49.3% (95% CI = 37.9%-60.7%). However, as the regression analysis shows (Figure S1 in the **Online Supplementary Document**), there WAS a slight increasing trend of the prevalence of myopia (equation of the regression line: myopia prevalence (%) = 0.00824 × midpoint of the study year group – 16.47958; *P* < 0.01).

**Table 3:** Subgroup analysis of refractive errors in Chinese children

	Myopia					High myopia				Hyperopia				Astigmatism		
	**N**	**% (95% CI)**	**Heterogeneity**		**N**	**%(95% CI)**	**Heterogeneity**		**N**	**%(95% CI)**	**Heterogeneity**		**N**	**% (95% CI)**	**Heterogeneity**	
			**I^2^ (%)**	***P***			**I^2^ (%)**	**P**			**I^2^ (%)**	**P**			**I^2^ (%)**	**P**
**Region:**																
Rural	13	27.1(26.7-27.5)	99.8	<0.001	6	0.9(0.3-2.9)	99.2	<0.001	6	4.0(1.3-11.4)	99.8	<0.001	8	9.9(5.9-16.0)	99.6	<0.001
Urban	15	51.2(50.8-51.5)	99.8	<0.001	10	3.0(2.3-3.8)	97.7	<0.001	11	5.4(3.4-8.4)	99.4	<0.001	8	20.6(15.0-27.5)	99.6	<0.001
**District:**																
SC	19	31.4(31.0-31.7)	99.9	<0.001	9	0.5(0.3-0.8)	94.6	<0.001	11	10.8(10.5-11.1)	99.4	<0.001	11	12.2(8.2-17.8)	99.6	<0.001
NC	15	55.1(54.9-55.3)	99.8	<0.001	11	4.6(3.6-5.7)	99.0	<0.001	7	27.8(27.1-28.6)	99.8	<0.001	5	18.5(12.7-26.1)	99.5	<0.001
HMT	5	53.0(52.6-53.4)	99.8	<0.001	4	4.9(3.5-6.9)	99.1	<0.001	2	5.3(4.9-5.8)	98.4	<0.001	2	35.7(20.4-54.6)	99.9	<0.001
**Study year:**																
-2000	4	49.3(37.9-60.7)	99.9	<0.001	3	5.7(4.0-8.1)	99.1	<0.001	2	3.2(2.1-4.9)	94.9	<0.001	3	29.4(16.0-47.6)	99.9	<0.001
2000-2010	13	36.5(32.6-40.5)	99.8	<0.001	4	3.2(2.4-4.2)	94.1	<0.001	9	4.1(2.2-7.4)	99.3	<0.001	8	9.5(5.2-16.7)	99.6	<0.001
2010-	23	36.6(31.3-42.4)	99.9	<0.001	15	2.1(1.5-2.9)	99.4	<0.001	7	5.9(3.4-9.9)	99.5	<0.001	6	18.3(13.0-25.1)	99.5	<0.001

N – number of studies, % - pooled prevalence, SC –Southern China, NC – Northern China, HMT – Hong Kong, Macau and Taiwan

Result of the meta-analysis of high myopia is presented in **Figure 2****,** Panel B. The pooled prevalence of high myopia was 2.8% (95% CI = 2.3%-3.4%). There was a higher prevalence of high myopia in urban areas vs rural areas: 3.0% (95% CI = 2.3%-3.8%) vs 0.9% (95% CI = 0.3%-2.9%); *P* < 0.001. For populations in different districts, HMT and Northern China had similar pooled prevalence: 4.9% (95% CI = 3.5%-6.9%) vs 4.6% (95% CI = 3.6%-5.7%), and Southern China still had the highest pooled prevalence: 0.5% (95% CI = 0.3%-0.8%), *P* < 0.001. Meta regression (Figure S2 in the **Online Supplementary Document**) shows that the high myopia prevalence has a weak decreasing trend (equation of the regression line: high myopia prevalence [%] = -0.01469 × midpoint of the study year group + 26.70921; *P* < 0.01).

### Prevalence of hyperopia

As shown in and **Figure 2****,** Panel C, pooled prevalence of hyperopia was 5.2% (95% CI = 3.1%-8.6%). Prevalence of hyperopia was higher in urban children than in rural children: 4.0% (95% CI = 1.3%-11.4%) vs 5.4% (95% CI = 3.4%-8.4%); *P* < 0.001. However, prevalence of hyperopia in HMT was lowest, while Northern China had the highest prevalence: 5.3% (95% CI = 4.9%-5.8%) vs 27.8% (95% CI = 27.1%-28.6%); *P* < 0.001. As for the subgroup analysis of study year, prevalence of hyperopia after the year 2010 was highest while prior to the year 2000 was lowest: 5.9% (95% CI = 3.4%-9.9%) vs 3.2% (95% CI = 2.1%-4.9%); *P* < 0.001. Result of the meta regression (Figure S3 in the **Online Supplementary Document**) shows an increasing trend of hyperopia prevalence (hyperopia prevalence (%) = 0.06933 × midpoint of the study year group – 141.49412; *P* < 0.01).

### Prevalence of astigmatism

**Figure 2**, Panel D, shows the meta-analysis results of astigmatism. The pooled prevalence of astigmatism was 16.5% ( 95% CI = 12.3%-21.8%). According to the subgroup analysis by region type, prevalence of astigmatism in urban areas was dramatically higher than in rural areas: 20.6% (95% CI = 15%-27.5%) vs 9.9% (95% CI = 5.9% 16.0%); *P* < 0.001. Prevalence of astigmatism in HMT (35.7%, 95% CI = 20.4%-54.6%) was highest, while prevalence in Southern China was lowest (12.2%,95% CI = 8.2%-17.8%); *P* < 0.001. Before the year 2000 the prevalence of astigmatism was highest and prevalence was lowest in the years 2000-2010 (29.4%, 95% CI = 16.0%-47.6%) vs 47.6% (95% CI = 5.2%-16.7%); *P* < 0.001. A decreasing trend was detected in the meta regression analysis, which is shown in Figure S4 in the **Online Supplementary Document** (astigmatism prevalence (%) = -0.06604 × midpoint of the study year group + 131.37988; *P* < 0.01).

### Publication bias and sensitivity analysis

According to the result of the Begg’s and Egger’s test, there was no publication bias detected for the prevalence of myopia, hyperopia and astigmatism (*P* > 0.05). We have also conducted the sensitivity analysis and the pooled prevalence RE did not change significantly compared with the initial results after removing each study sequentially, suggesting good homogeneity of the included studies.

## DISCUSSION

In this study, 41 studies conducted in China were included for this meta-analysis and the pooled prevalence of myopia, high myopia, hyperopia, astigmatism in Chinese children were 38.0%, 2.8%, 5.2%, 16.5%, respectively. The prevalence of RE varied across different districts, region-type and period.

Compare with previous meta-analysis that reported the overall prevalence of myopia and high myopia in Chinese children from 1998 to 2016 [10], the prevalence of myopia and high myopia remains in a high level (for myopia:37.7% vs 38%, for high myopia: 3.1% vs 2.8%), which suggest that much more efforts should be made in future to prevent and control myopia in China. Subgroup analysis in this study showed that the prevalence of myopia in urban areas is dramatically higher than that of rural areas in China. Reasons that lead to the higher prevalence of myopia in urban areas are varied, such as less outdoor activities and high academic stress [7,8]. Population in HMT have higher myopia prevalence in this study, which is comprehensible since these districts are highly-urbanized. According to the regression analysis, there is an increasing trend of prevalence of myopia. However, as shown in the subgroup analysis by study year, the prevalence of myopia and high myopia before the year 2000 are higher than the prevalence after the year 2010, which may be because there are fewer studies reporting prevalence before 2000 and many of them were conducted in Taiwan, a city with high prevalence of refractive errors [10,26,27,50].

It is worth noting that the increasing trend of myopia has a slowdown trend compared with the previous meta-analysis which showed a higher slope in regression analysis (1.086 vs 0.008) and predicted that the estimated prevalence of myopia in 2050 was 84% [10]. This might reflected that the myopia prevention and control strategies in China [52-54], including lightening student’s schoolwork burden, encouraging children to spend more time outdoors and wider use of Orthokeratology, has made some achievements in the past years.[55] Especially, the Sports for All National Strategy carried out by the government in the thirteenth Five-Year Plan of China (2016-2020), may have played an important role in myopia prevention and control in China [56,57].

Comparing the prevalence of myopia in different countries, we found that prevalence of myopia and high myopia in Chinese children is significantly higher than in many other countries. For example, the prevalence of myopia ranges from 6.1% in Morocco [58], 4.0% in Middle East [59], to 0.8% in Laos [60]. As for high myopia, the prevalence was 1.0% in Korea [61], and 1.4% in India [62]. Possible explanations for this difference may lie in several aspects. First, Pan et al. revealed that factors such as higher educational level and exposure to an intensive schooling system at an early age, especially in countries such as China, are positively associated with myopia [8]; second, ethnic difference that leads to a higher prevalence in China are already discussed in many articles [7,10,63]. However, whether the difference between different ethnicities is caused by inter-ethnic differences in the genetic predisposition to myopia or culture-specific environmental factors still remains unclear [64].

In terms of hyperopia, as the first meta-analysis reporting prevalence of hyperopia in Chinese Children, our results show a higher prevalence was detected in urban compared to rural areas which is inconsistent with the study performed in India which showed children in rural areas were more likely to develop hyperopia than those in urban areas [65]. In our study, the higher prevalence of hyperopia in urban areas may lie in that the mean age of participants in urban areas are younger than participants in rural areas and a previous study has revealed that there is an inverse association between prevalence of hyperopia and age [66]. As for regression analysis of hyperopia, we found an increasing trend, which could be explained by the physical education reform of China (Sports for All National Strategy) which encourage children to spend more time on outdoor sports, and the policies for myopia prevention and control mentioned above [53,54,56,57].

When comparing the prevalence of hyperopia with other countries and regions, prevalence of hyperopia is relatively low in China which is similar to other east Asian countries. For example, the prevalence of hyperopia is 1.5% in Singapore and 6.2% in Korea [61,67]. In contrast, prevalence is higher in western countries, from 13.1% in Poland to 14.7% in Northern Ireland [68,69]. Both environmental factors and ethnic and genetic factors may contribute to the low prevalence of hyperopia in China. For the environmental factors, as is mentioned above, spending more time outdoors and living in rural areas often leads to hyperopia while children in China tend to spend less time outdoors and more time in near work [65,70]. As for the ethnic factor of hyperopia, the CLEERE study reported that Caucasians had the highest prevalence of hyperopia while Asians have the lowest prevalence [63,66]. According to the meta-analysis by Hashemi et al [6], genetic and ethnic factors could play a more prominent role in hyperopia.

The prevalence of astigmatism in Chinese children is 16.5% and highest prevalence was seen in HMT (35.7%), which is higher than many countries. For example, prevalence was only 5.4% in India, 6.7% in Australia and 9% in Laos [60,62,71]. As is mentioned earlier, near work is one of the major reason that leads to astigmatism, and the high stress on academic performance may contribute to the high prevalence of astigmatism in China [72,73]. Ethnicity also plays an important part in the prevalence of astigmatism. As is reported in the CLEERE study, Asians and Hispanics had the highest prevalence of astigmatism [63], which may be explained by the anatomy of Asian eyes (tight eyelids and narrow palpebral apertures) [74]. When compared our results with the study conducted in Taiwan, which reported a prevalence of astigmatism was 42.5% in 1995 and 51% in 2000, an obvious decreasing trend was suggested. A reasonable explanation might be the perform of series policies, including the myopia prevent strategies mentioned above [72]. In the subgroup analysis by region, a higher astigmatism prevalence was detected in urban regions vs rural regions. One explanation could be urban children are engaging in more near work, and as a former study reported not only can near work cause myopia but it is also likely to increase the risk of astigmatism [72]. In the subgroup analysis by district, the highest astigmatism prevalence was found in HMT, which are highly urbanization areas, which is consistent with subgroup analysis of region-type.

This study has several limitations. First, few studies were prior to the 2000 or in remote provinces which will affect the precision of the results to a certain extent. Second, not all the studies used the same definition of RE which might influence the result in some extent. Third, out of 40 studies, 9 of them did not perform cycloplegia for refractometry which might also influence the result. Nevertheless, to the best of our knowledge, this is the first meta-analysis to report the overall prevalence and time trend analysis of refractive errors and its sub-classifications in Chinese children. Additionally, this article includes numerous studies throughout China, covering a large population with a wide geographical distribution.

## CONCLUSION

The pooled prevalence of myopia, high myopia, hyperopia, astigmatism in Chinese children are 38.0%, 2.8%, 5.2%, 16.5%, respectively. Urban children are more vulnerable to RE (especially myopia) than rural children. Children living in HMT have a higher prevalence of myopia, high myopia and astigmatism than children in mainland China. There is an increasing trend for prevalence of myopia and hyperopia while there is a decreasing trend for prevalence of high myopia and astigmatism in Chinese children. Considering the large magnitude of refractive errors, more attention should be paid to RE prevention and treatment strategy development in China.

## Additional material

Online Supplementary Document
