# Supplementary material for: Prevalence and time trends of refractive error in Chinese children: A systematic review and meta-analysis
Source: J Glob Health. 2021 Jul 17;11:08006. doi: 10.7189/jogh.11.08006 (PMC8285767; doi:10.7189/jogh.11.08006)

Figure S1. Association between the prevalence of myopia in Chinese children with the study year.

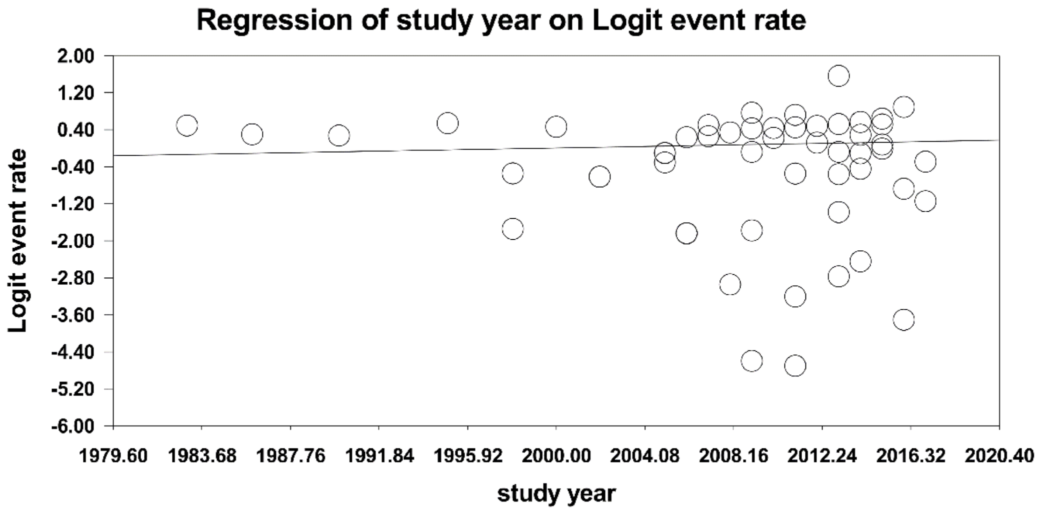

Figure S2. Association between the prevalence of high myopia in Chinese children with the study year.

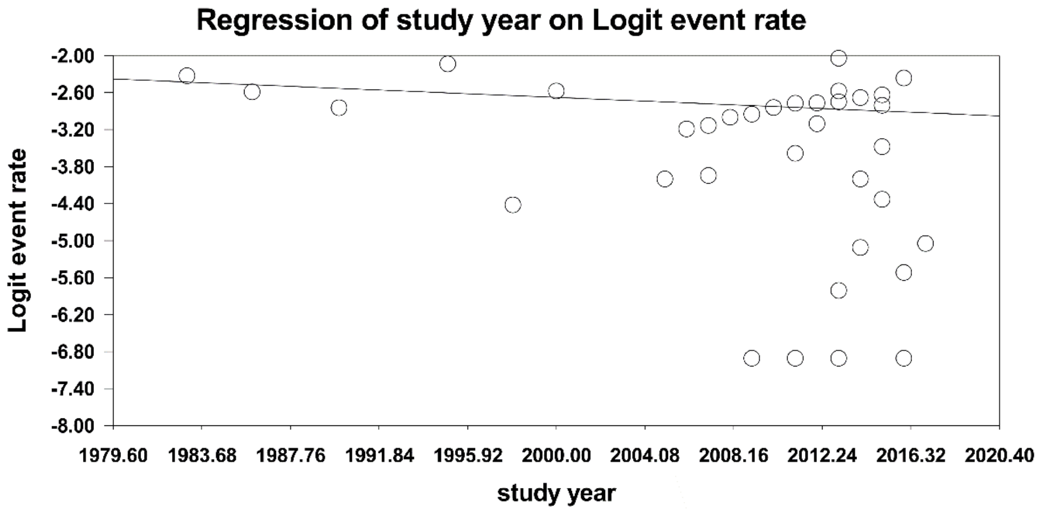

Figure S3. Association between the prevalence of hyperopia in Chinese

children with the study year.

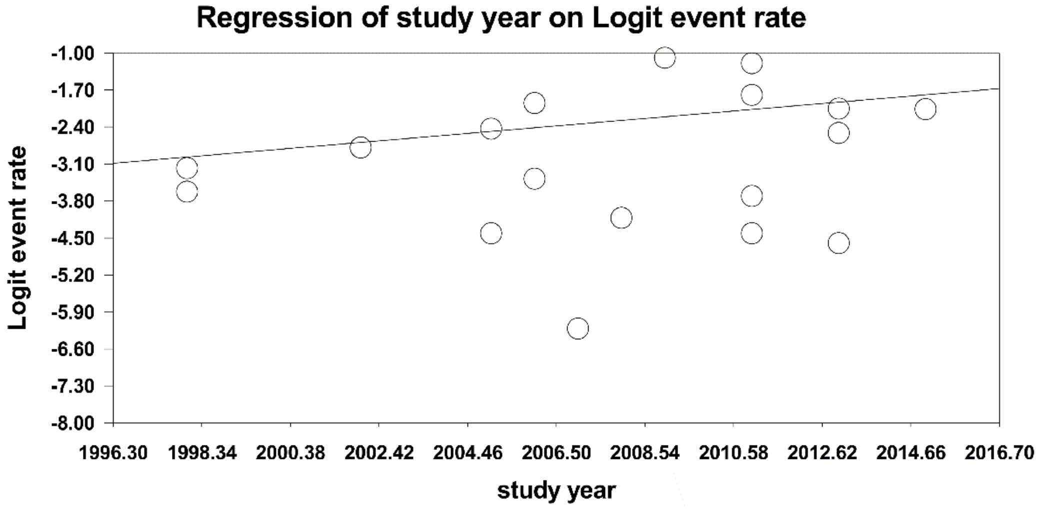

Figure S4. Association between the prevalence of astigmatism in Chinese children with the study year.

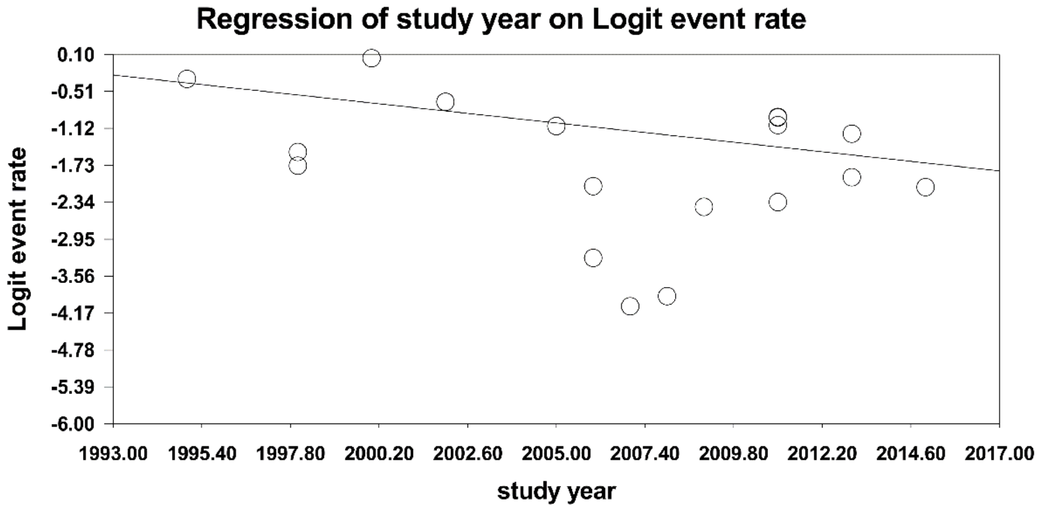

Supplement: Online Supplementary Document [file jogh-11-08006-s001.pdf]
